# Economical droplet-based microfluidic production of [^18^F]FET and [^18^F]Florbetaben suitable for human use

**DOI:** 10.1038/s41598-021-99111-4

**Published:** 2021-10-19

**Authors:** Ksenia Lisova, Jia Wang, Tibor Jacob Hajagos, Yingqing Lu, Alexander Hsiao, Arkadij Elizarov, R. Michael van Dam

**Affiliations:** 1 Crump Institute for Molecular Imaging, University of California Los Angeles, Los Angeles, CA USA; 2 Department of Molecular and Medical Pharmacology, University of California Los Angeles, Los Angeles, CA USA; 3 Physics in Biology and Medicine Interdepartmental Graduate Program, University of California Los Angeles, Los Angeles, CA USA; 4 Bioengineering Department, University of California Los Angeles, Los Angeles, CA USA; 5 Trace-Ability, Inc., Van Nuys, CA USA; 6 SOFIE, Inc., Dulles, VA USA

**Keywords:** Biomedical engineering, Automation, Diagnostic markers

## Abstract

Current equipment and methods for preparation of radiopharmaceuticals for positron emission tomography (PET) are expensive and best suited for large-scale multi-doses batches. Microfluidic radiosynthesizers have been shown to provide an economic approach to synthesize these compounds in smaller quantities, but can also be scaled to clinically-relevant levels. Batch microfluidic approaches, in particular, offer significant reduction in system size and reagent consumption. Here we show a simple and rapid technique to concentrate the radioisotope, prior to synthesis in a droplet-based radiosynthesizer, enabling production of clinically-relevant batches of [^18^F]FET and [^18^F]FBB. The synthesis was carried out with an automated synthesizer platform based on a disposable Teflon-silicon surface-tension trap chip. Up to 0.1 mL (4 GBq) of radioactivity was used per synthesis by drying cyclotron-produced aqueous [^18^F]fluoride in small increments directly inside the reaction site. Precursor solution (10 µL) was added to the dried [^18^F]fluoride, the reaction chip was heated for 5 min to perform radiofluorination, and then a deprotection step was performed with addition of acid solution and heating. The product was recovered in 80 µL volume and transferred to analytical HPLC for purification. Purified product was formulated via evaporation and resuspension or a micro-SPE formulation system. Quality control testing was performed on 3 sequential batches of each tracer. The method afforded production of up to 0.8 GBq of [^18^F]FET and [^18^F]FBB. Each production was completed within an hour. All batches passed quality control testing, confirming suitability for human use. In summary, we present a simple and efficient synthesis of clinically-relevant batches of [^18^F]FET and [^18^F]FBB using a microfluidic radiosynthesizer. This work demonstrates that the droplet-based micro-radiosynthesizer has a potential for batch-on-demand synthesis of ^18^F-labeled radiopharmaceuticals for human use.

## Introduction

Diagnostic radiopharmaceuticals (tracers) used in positron-emission tomography (PET) imaging enable a wide range of research and clinical applications including cancer diagnostics and tumor severity grading^1–3^, evaluation of response to cancer therapy^4,5^, diagnostics of neurodegenerative disease^6–8^, cardiac function assessment^9,10^, drug development^11–13^ and development of novel gene- and cell-based therapies^14–16^. Of thousands of developed tracers to probe different biological targets and processes^17,18^, only very few are routinely available. The complexity and high cost of manufacturing short-lived PET tracers has led to a centralized production model, where large batches of the tracers are produced in radiopharmacies, and then batches (and costs) are split to be distributed to multiple PET centers. Since a significant demand is needed to justify the high costs of establishing and performing the syntheses using conventional instrumentation and facilities, availability of specialized tracers is limited.

Recent advancements in PET radiochemistry directed at development of batch-on-demand systems are creating new possibilities to expand availability of diverse diagnostic radiopharmaceuticals at low cost. Microfluidics offers a promising approach to enable economic production of one to a few patient doses due to advantages such as reduced (10–100×) reagent consumption, faster reaction kinetics, improved product yields, and reduced equipment footprint and shielding size^19–24^. Numerous reports have established the feasibility of synthesizing various radiopharmaceuticals using microfluidic synthesizers. However, due to the disparity between the volume of radioisotope solutions (~ 1 mL) and reaction volumes of microscale systems (as low as 1–10 s of µL), relatively small amounts of product activity have been acquired, suitable only for preclinical imaging^19,24,25^. Nevertheless, clinically-relevant quantities of various diagnostic radiopharmaceuticals has been produced with such microscale systems: [^13^N]NH_3_^26^, [^68^Ga]Ga-PSMA-11^27^, [^89^Zr]Zr-DFO-Trastuzamab^28^, [^18^F]FDG^29,30^, [^18^F]FET^31^, [^18^F]fallypride^32,33^, [^18^F]FT807^34^, [^18^F]FPEB^35^, [^18^F]FLT^36^ and [^18^F]FMISO^36,37^. A summary of reports of the ^18^F-labeled ones is included in Table 1.

**Table 1:** Literature reports of microfluidic production of ^18^F-labeled radiopharmaceuticals with sufficient quantities for human PET. *N.R.* not reported.

Reference	This work		Wang et al.^33^	Lebedev et al.^32^	Frank et al.^29^	Liang et al.^34^	Liang et al.^35^	Zheng et al.^37^	Akula et al.^36^		Awasthi^30^	Iwata et al.^31^
Microfluidic synthesis platform	Droplet-based radiosynthesizer		Droplet-based radiosynthesizer	PEEK/pDCPD chip with syringe-type microvalves	GE ISAR	Advion NanoTek	Advion NanoTek	Advion NanoTek	Advion NanoTek		ABT BG75	Disposable glass vials with a fused 300-μL insert
Synthesis format	Batch		Batch	Batch	Batch	Flow	Flow	Flow	Flow		Batch	Batch
Tracer(s) produced	[^18^F]FET	[^18^F]FBB	[^18^F]Fallypride	[^18^F]Fallypride	[^18^F]FDG	[^18^F]T807	[^18^F]FPEB	[^18^F]FMISO	[^18^F]FLT	[^18^F]FMISO	[^18^F]FDG	[^18^F]FET
Starting activity (GBq)	2.7 ± 0.4 (n = 3)	3.2 ± 0.8 (n = 6)	Up to 41	Up to 111	Up to 170	16.1 ± 4.4 (n = 3)	80.9	~ 5.6	13^b^	13^b^	~ 1.9	Up to 6
Product activity (GBq)	0.6 ± 0.2 (n = 3)	0.5 ± 0.2 (n = 6)	Up to 7.2	N.R	> 100	4.4 ± 0.1 (n = 3)	1.7 ± 0.4 (n = 3)	1.5–1.9	2.2	2.1	0.4–0.6	Up to 4^c^
Molar activity (GBq/µmol)	420 ± 50 (n = 3)	480 ± 160 (n = 5)	Up to 270	N.R	N.R	220 ± 50 (n = 3)	160 ± 10 (n = 3)	120 ± 30 (n = 4)	> 74	> 74	N.R	480 ± 130 (n = 7)
Synthesis time (min)	60	60	50	45	< 25	< 100	75	106 ± 11 (n = 15)	77	53	40–60	50
Precursor consumed (nmol)	60	80	616	1940	N.R	1560	21,500	940	24,100	11,800	N.R	177
Reaction volume (µL)	10	10	8	50	650^a^	400	1000	200	2000	2000	N.R	60
QC testing reported?	Yes	Yes	Yes	Yes	No	Yes	Yes	Yes	No	No	Yes	No
Used in patients?	No	No	No	Yes	No	Yes	No	Yes	No	No	No	No

^a^Precise reaction volume was not reported, but the total reactor size was 650 µL.

^b^Total activity used for [^18^F]FLT and [^18^F]FMISO sequential syntheses combined is reported, approximately half used in each synthesis.

^c^Estimated from reported crude yield value for 6 GBq starting activity and assuming 50 min synthesis time.

Microfluidic reactors can be classified in two categories: continuous-flow synthesizers, where the reaction volume is flowed through a microchannel or capillary, and batch-mode synthesizers, that contain a fixed reaction volume confined within a miniature reaction chamber^38^ or within an isolated droplet^19,21^. In the continuous-flow systems, radioisotope and precursor solution streams are mixed prior to entering the heated reaction zone. Scaling of the product activity can be easily achieved by increasing radioisotope volume and also a corresponding increase of precursor solution volume, or by concentrating the isotope prior to synthesis. The first PET tracer suitable for human use produced in a microfluidic continuous-flow reactor was demonstrated using the commercial NanoTek radiosynthesizer (Advion, Inc., Ithaca, NY): Liang et al*.* reported starting activities of up to 170 GBq, and the synthesis of 1.7 GBq of [^18^F]FPEB^35^. In a separate report, Liang et al*.* also reported the synthesis of 4.4 GBq batches of [^18^F]T807 (each with 16 GBq starting activity), for the first time administering the tracer produced by continuous-flow reactor to a human subject^34^. Using the same system, Zheng et al*.* reported the synthesis of up to 1.9 GBq of [^18^F]FMISO (with 5.6 GBq starting activity) for use in clinical research, and Akula et al*.* reported the sequential production of 2 tracers [^18^F]FLT and [^18^F]FMISO in ~ 2 GBq quantities, each from 13 GBq of starting radioactivity^36^. Despite impressive scalability, continuous-flow reactors use relatively large total reaction volumes (100 s of µL), with 100 s of µg of precursor to prepare these clinical-scale batches, and require an extended time for the initial [^18^F]fluoride preparation step^19^.

Batch reactors offer a drastic reduction in precursor consumption (< 100 µg) which is independent of the amount of loaded radioisotope. However, to produce clinically-relevant quantities of the radiopharmaceutical in these tiny reaction volumes, pre-concentration of [^18^F]fluoride is necessary. By adapting conventional azeotropic drying to the ISAR platform (GE Global Research Europe), Frank et al*.* reported the synthesis of > 100 GBq of [^18^F]FDG using starting activity up to 170 GBq^29^. Using the BG75 system (ABT Molecular Imaging, Knoxville, TN) system, which integrates into a small cyclotron, Awasthi et al*.* reported synthesis of [^18^F]FDG from 1.9 GBq of starting activity, concentrated via azeotropic drying in the reaction vessel, to produce single, injectable human doses (0.4–0.6 GBq)^30^. Iwata et al*.* developed a trap-and-release process using a combination of commercially-available cation- and anion-exchange cartridges to trap 1 mL of cyclotron-produced [^18^F]fluoride (up to 6 GBq) and release it in a 0.2 mL methanolic solution that could be rapidly evaporated in a small vial designed for 5–20 µL subsequent reaction to produce [^18^F]FET^31^. The first human images obtain using a microfluidically-produced PET tracer were synthesized in a 50 µL batch reactor platform, in this work Lebedev et al*.* performed an upstream trap-and-release process on a miniature QMA cartridge to concentrate a full cyclotron-target volume of [^18^F]fluoride (e.g. ~ 100 GBq in 2 mL) into < 45 µL. This could be loaded into the reactor and evaporatively dried, enabling the synthesis of up to 38 GBq of [^18^F]fallypride^32^. Chao et al*.* designed a standalone radioisotope concentrator system based on a similar mini-QMA approach, capable of concentrating milliliter-scale [^18^F]fluoride batches into ~ 12 µL volume^39^. The device was subsequently integrated with an automated droplet radiosynthesizer, to concentrate starting activities of up to 41 GBq. Production of quantities of formulated [^18^F]fallypride up to 7.2 GBq were demonstrated^33^.

While these methods are all effective, integration with any type of concentrator increases system complexity and synthesis time, and, except for the Iwata et al*.* method^31^, requires optimization of base quantities used during the [^18^F]fluoride elution process to avoid adversely affecting the downstream synthesis. Instead, a simpler sequential drying approach can be used with droplet reactors, in which the initial radioisotope solution is subdivided into smaller portions each added and then rapidly evaporated (due to the high surface to volume ratio of small volumes), to build up the amount of activity in the reaction site. For example, Chen et al*.* heated a 200 µL droplet of [^18^F]fluoride solution on an open surface until it shrunk to 5 µL and then transported this concentrated droplet into an electrowetting-on-dielectric (EWOD) radiosynthesis chip for completion of the drying step^40^. We later demonstrated the possibility for rapid concentration by evaporation by sequentially loading to 2 µL portions to a pre-heated chip^41^. Since each drying iteration takes time, there is a practical limit on the volume/amount of radioactivity that can be concentrated, but evaporation is quite quick for modest batches. Drying of volumes in a range of a few hundred microliters is feasible, and can provide enough starting radioactivity for synthesis of clinically-relevant batches^19^. In this work, we leverage the larger volume of the reaction site of the surface-tension trap (STT) design chip^42^ compared to the passive transport (PT) design chip^41^, and concentrate [^18^F]fluoride by loading and drying it in 30 µL increments. The goal of the present work is to demonstrate that tracers other than [^18^F]fallypride can be produced at clinically-relevant scales using this simple approach for [^18^F]fluoride concentration and thus with a simple overall apparatus.

Previously, we reported the production of the amino acid PET tracer *O*-(2-[^18^F]fluoroethyl-)-l-tyrosine ([^18^F]FET)^43^ and the stilbene derivative 4-[(E)-2-(4-{2-[2-(2-[^18^F]fluoroethoxy)ethoxy] ethoxy}phenyl)vinyl]-*N*-methylaniline ([^18^F]florbetaben, [^18^F]FBB, Neuraceq™, BAY-949172) in a droplet reactor, observing, for each, significant advantages compared to conventional synthesis methods. [^18^F]FET PET assesses amino acid transport and is used for glioma differentiation from non-neoplastic lesions and glioma grading^44^, while [^18^F]FBB PET visualizes amyloid plaques and aids in diagnosis of Alzheimer’s disease^8^. Using the simplified [^18^F]fluoride concentration method described above, we adapted the previous synthesis methods to scale up the production of [^18^F]FET and [^18^F]FBB to amounts sufficient for clinical use (i.e. one to a few human doses). Furthermore, quality control (QC) testing was performed to ensure the tracer batches meet the necessary specifications for clinical use. Some of the QC tests were performed using the Tracer-QC automated testing platform (Trace-Ability, Inc., Van Nuys, CA, USA), showing the successful integration of a novel compact microfluidic radiosynthesis platform and a modern benchtop QC testing platform, and demonstrating the possibility for clinically-relevant radiotracer production with an overall compact, user-friendly system.

## Materials and methods

### Materials

#### Reagents

No-carrier-added [^18^F]fluoride was produced by the ^18^O(p, n)^18^F reaction from [^18^O]H_2_O (84% isotopic purity, Zevacor Pharma, Noblesville, IN, USA) in an RDS-112 cyclotron (Siemens; Knoxville, TN, USA) at 11 MeV using a 1 mL tantalum target with havar foil. Acetonitrile (MeCN; anhydrous, 99.8%), methanol (MeOH; anhydrous, 99.8%), 2,3-dimethyl-2-butanol (thexyl alcohol (TA); 98%), ethanol (EtOH; 200 proof, > 99.5%), hydrochloric acid (HCl; 1 M), dimethylsulfoxide (DMSO; 98%), deionized (DI) water, and polyethylene glycol 400 (PEG 400), Kryptofix 222 (K_222_) and potassium carbonate (K_2_CO_3_) were purchased from Millipore Sigma (St. Louis, MO, USA). Sodium phosphate dibasic (Na_2_HPO_4_–7H_2_O) and sodium phosphate monobasic (NaH_2_PO_4_H_2_O) were purchased from Fisher Scientific (Thermo Fisher Scientific, Waltham, MA, USA). Saline (0.9% sodium chloride injection, USP) was obtained from Hospira Inc. (Lake Forest, IL, USA). Tetrabutylammonium bicarbonate 0.075 M (TBAHCO_3_, > 99%), (2S)-*O*-(2′-tosyloxyethyl)-*N*-trityl-tyrosine-tert-butyl ester (TET; > 95%) (FET precursor), *O*-2-fluoroethyl-l-tyrosine (FET-HCl; > 95%) (FET reference standard) were purchased from ABX GmbH (Radeberg, Germany). ([Methanesulfonic acid 2-{2-[2-(4-{2-[4-(tert-butoxycarbonyl-methyl-amino)-phenyl]-vinyl}-phenoxy)-ethoxy]-ethoxy}-ethyl ester) (FBB precursor) and (4-[(E)-2-(4-{2-[2-(2-[^18^F]fluoroethoxy) ethoxy] ethoxy} phenyl) vinyl]-*N*-methylaniline) (FBB reference standard) were generously provided by Life Molecular Imaging GmbH as a part of [^18^F]Florbetaben synthesis kits (Life Molecular Imaging GmbH, Berlin, Germany). Dry scavenger (to prevent radiolysis), consisting of sodium ascorbate with l-ascorbic acid (87:13 w/w), was also obtained from the same [^18^F]Florbetaben kits. All reagents were used as received without further purification. Ultrapure 18 MΩ H_2_O was obtained from a Milli-Q Integral 3 purification system (Millipore Sigma, St. Louis, MO, USA).

Stock K_222_/K_2_CO_3_ solution (for [^18^F]FBB synthesis) was prepared by first making an aqueous 61 mM K_2_CO_3_ mixture and adding K_222_ to reach 85 mM concentration. Stock solutions were prepared for FET precursor [6 mM in MeCN:TA 1:1 (v/v)], FBB precursor (8 mM in DMSO), and for [^18^F]FET collection solution [1:1 MeOH:H_2_O (v/v)] and [^18^F]FBB collection solution [1:1 MeCN: H_2_O (v/v)]. Acid mixture used for deprotection in both syntheses was made by mixing MeCN and HCl 1:1 (v/v). Scavenger solution for [^18^F]FBB was prepared either at 33 mg/mL or 10 mg/mL in H_2_O. Formulation dilution solution for [^18^F]FBB contained 39 mg/mL of dry scavenger in a 4:13 (v/v) mixture of PEG 400 and H_2_O.

### Automated droplet synthesizer

**Figure 1:**
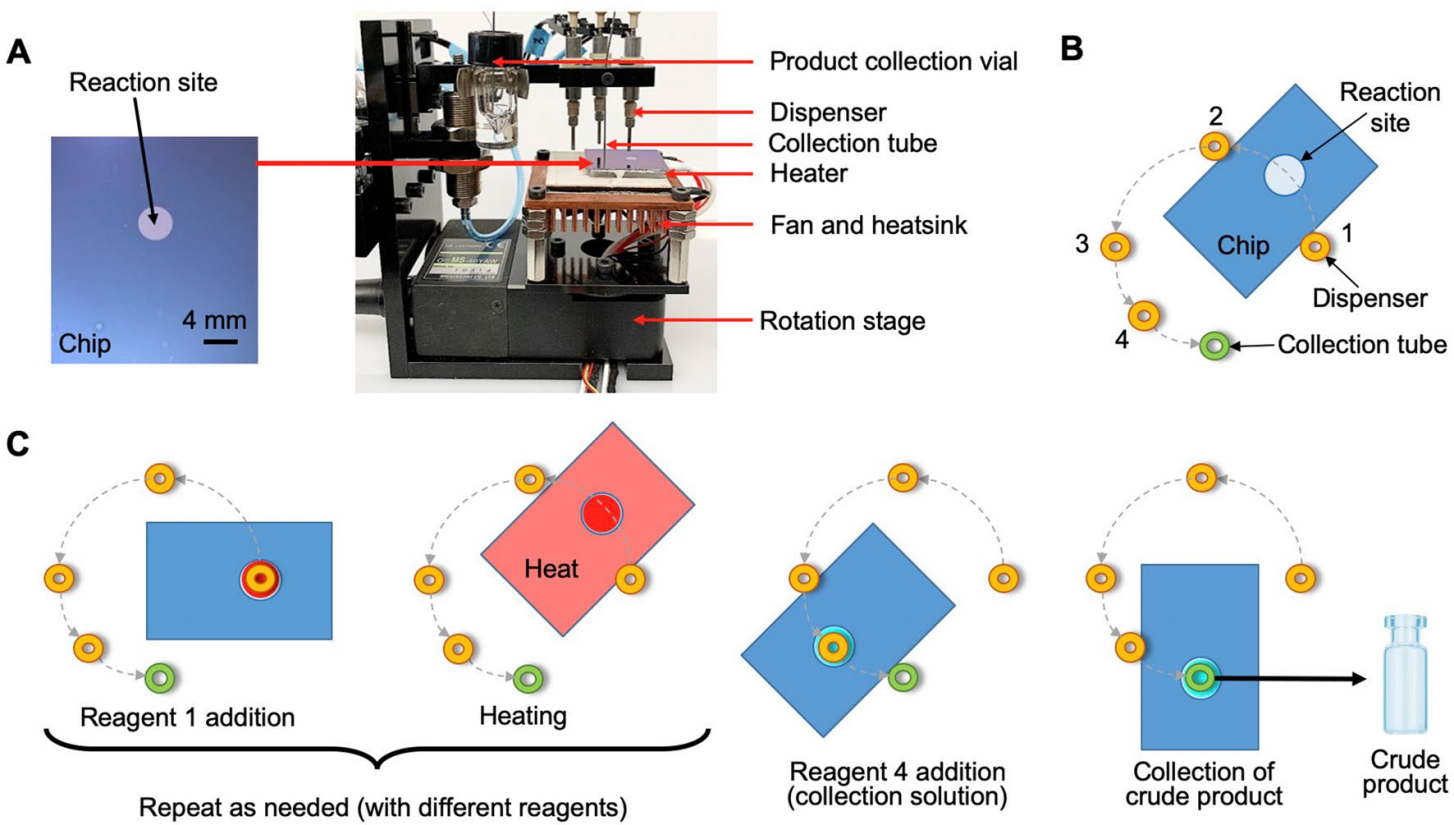
(**A**) Photographs of a disposable reaction chip (left) and automated droplet synthesizer (right). (**B**) Top view schematic of dispenser arrangement for a multi-step droplet synthesis. (**C**) Simplified schematic showing position of rotating platform during various steps of a typical radiosynthesis (reagent addition, heating, and collection of crude product).

Radiosyntheses were performed in a droplet format on the surface of disposable silicon-Teflon chips (surface-tension trap (STT) chips) and using an automated radiosynthesizer system to dispense reagents and recover syntheses products (Fig. 1A)^42^. Each 25.0 × 27.5 mm^2^ chip was coated with hydrophobic Teflon layer with an etched hydrophilic circular reaction site (4 mm diameter), which acted as a surface-tension trap to confine reagents during the multi-step radiosynthesis. The details of the STT chip fabrication were previously reported^42^. The chip was placed atop a heater that can rotate, and reagents were delivered by piezoelectric dispensers arranged in a circular pattern above the chip. Dispensers were calibrated and primed before use as described previously^41^. The operation of this synthesizer is illustrated in Fig. 1B.

For synthesis with high (up to multi-GBq) starting activities, the desired quantity of [^18^F]fluoride was pre-mixed with either TBAHCO_3_ (113 nmol) for synthesis of [^18F^]FET or K_222_/K2CO_3_ (383/275 nmol) for synthesis of [^18^F]FBB, then dispensed and dried on chip in portions of up to 30 µL at a time (the maximum capacity of the reaction site). Up to 4 droplets were used to load activities in the range 0.02–4 GBq.

**Figure 2:**
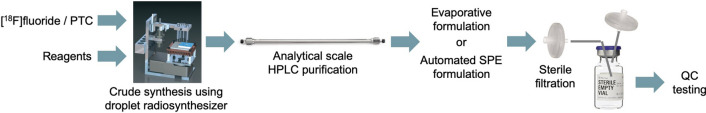
Tracer preparation scheme. *PTC* phase transfer catalyst, *SPE* Solid-phase extraction.

Following crude synthesis of the tracers, purification was achieved using analytical-scale HPLC with a tracer-specific method reported previously^43,45^. Then the tracers were reformulated either by evaporation and resuspension ([^18^F]FET)^43^, or automated solid-phase extraction (SPE) ([^18^F]FBB)^45^ (Fig. 2) followed by sterile filtration.

### [^18^F]FET synthesis

The production of [^18^F]FET was performed using identical reaction conditions as previously reported for manual droplet-based synthesis^43^ adapted from a conventional 2-step synthesis route^46,47^.

**Figure 3:**
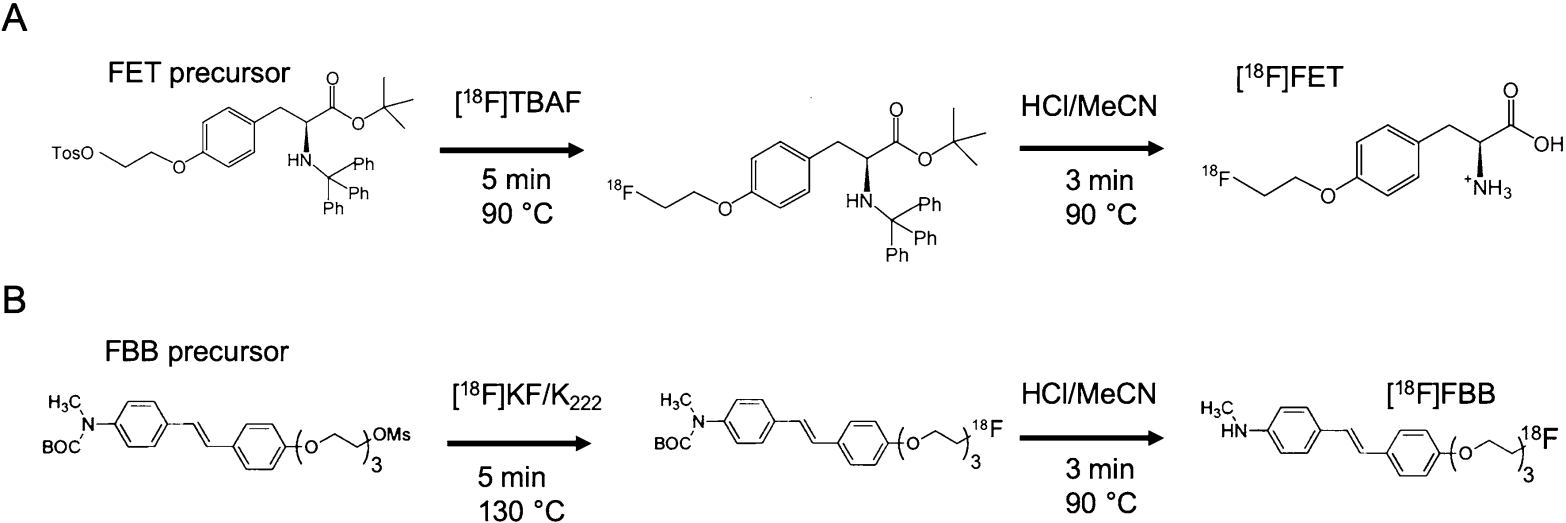
Synthesis routes for (**A**) [^18^F]FET and (**B**) [^18^F]FBB.

The synthesizer was set up by loading stock solutions into reagent dispensers as indicated in Supplementary Table S1. As the last setup step, the desired activity of [^18^F]fluoride was mixed with 1.5 μL of 0.075 M TBAHCO_3_ and loaded in the corresponding dispenser. The 2-step (fluorination and deprotection) crude synthesis was carried out as shown n Fig. 3A. The [^18^F]fluoride/TBAHCO_3_ solution was loaded 30 μL at a time, each droplet dried at 100 °C for 1.5 min. To the dried residue, precursor solution (10 µL) was added and the radiofluorination step was performed (5 min, 90 °C). Acid mixture was then added to perform deprotection (3 min, 90 °C). 20 µL was added at the beginning, and another 20 µL was added after 1.5 min. The crude product was recovered with FET collection solution (4 × 20 µL). To obtain purified [^18^F]FET, the crude collection mixture was diluted with 100 µL water (to lower MeCN concentration, improve separation quality and reduce losses during sample transfer) and injected into analytical radio-HPLC for purification (conditions described below). The [^18^F]FET peak was collected in a pyrex vial (WHEATON^®^ V vial 5 mL, Millville, NJ, USA), evaporated to dryness in an oil bath at 120 °C and resuspended in 5 mL of sterile saline. The formulated product was sterile filtered (13 mm diameter, 0.22 mm pore size, PVDF membrane; Fisherbrand™, Waltham, MA, USA) into a sterile product vial (2 mL, ALK, Denmark) and samples taken under aseptic conditions for QC testing. Clinical-scale batches were prepared with at least 2 GBq of starting activity.

### [^18^F]FBB synthesis

Automated production of [^18^F]FBB in droplet format, adapted from a 2-step conventional synthesis route Fig. 3B using a Boc-protected precursor^48^, was previously reported^45^. In this work the volume of precursor solution was increased from 10 to 15 μL to reduce sensitivity of the reaction performance^45^ in case of dispensing errors associated with the viscous DMSO-based precursor solution.

The configuration of dispensers is described in Supplementary Table S1. The desired activity of [^18^F]fluoride was mixed with 4.5 μL of K_222_/K_2_CO_3_ stock solution and dispensed 30 μL at a time, with each droplet dried at 100 °C for 1.5 min. To the dried residue, precursor solution (10 or 15 µL) was added, and then the chip was heated for 5 min at 130 °C to perform radiofluorination of the precursor. Then, the acid solution was added (20 µL at t = 0, and another 20 µL at t = 1.5 min) to remove protecting groups (5 min, 90 °C). The crude product was recovered with FBB collection solution (4 × 20 µL) into a vial pre-filled with 64 µL of 33 mg/mL scavenger solution, diluted with 50 µL H_2_O, and purified via analytical HPLC. The purified product was formulated via SPE using an automated system^45^, from where it was eluted in ethanol and diluted with formulation dilution solution to achieve 15% EtOH concentration in a final volume of 5 mL, and sterile filtered (Whatman^®^, Anotop^®^ 10 mm diameter, 0.02 µm pore size; Cytiva, Marlborough, MA, USA). Samples were taken for QC testing. Batches intended for QC testing used at least 2 GBq starting activity. In case of samples analyzed with the Tracer-QC system, the elution step during formulation was performed with 150 µL EtOH, and the final formulated volume was 1 mL.

### Analytical methods

A calibrated ion chamber (CRC 25-PET, Capintec, Florham Park, NJ, USA) was used to perform radioactivity measurements. Radioactivity recovery was determined by dividing radioactivity of collected crude product by the amount of starting activity (correcting for decay). Fluorination efficiency was determined from radio-TLC as a percentage of desired product in the crude product. Crude radiochemical yield (crude RCY) was calculated by multiplying radioactivity recovery and fluorination efficiency. Overall RCY is a ratio of final formulated product activity to the starting activity. Molar activity was quantified based on isolated product radioactivity collected after HPLC purification and area under the corresponding UV peak of the purification chromatogram converted to molar quantity using a calibration curve.

Fluorination efficiency was determined via radio-thin-layer chromatography (radio-TLC). Radio-HPLC analysis and purification were performed on an analytical-scale HPLC system. These methods were reported previously, and are summarized in the Supplementary Sect. 2.

### Quality control testing

Quality control tests were performed on 3 consecutive batches of [^18^F]FET and 3 consecutive batches of [^18^F]FBB. Details of conventional quality control testing are described in the Supplementary Sect. 3. An additional 3 batches of [^18^F]FBB were prepared and transported to Trace-Ability, Inc. (Van Nuys, CA, USA), and tested using an automated QC testing system (Tracer-QC, Trace-Ability, Inc.).

**Figure 4:**
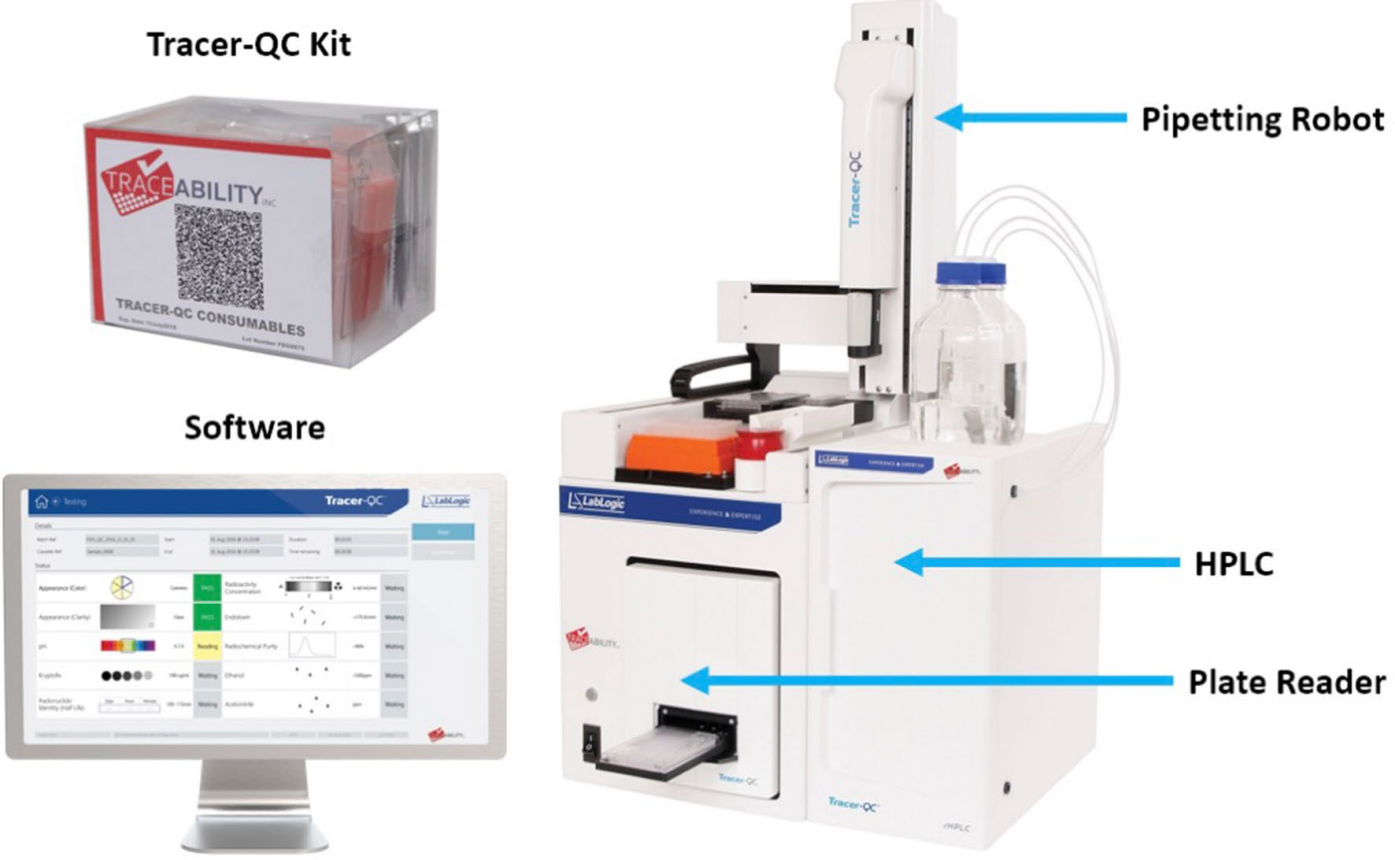
Components of the Tracer-QC platform.

This platform enables complete automation of PET tracer QC and comprises a plate reader, liquid handler and HPLC integrated into a single system that operates with disposable test kits (Fig. 4)^49^. To operate the system, the user installs the kit, initiates the program, delivers the sample, triggers the analysis and collects the report. After the process is complete and the used kit is removed, the system is ready for the next analysis without any further preparation. Table 2 summarizes the tests developed for FBB with comparison to conventional test methods. These tests have been developed and validated individually and then merged into an integrated protocol for automated execution. They have been subsequently verified or re-validated as suitable for QC testing of [^18^F]FBB produced on the miniaturized platform. The effects of the unique composition of [^18^F]FBB resulting from such syntheses were studied and reflected in the method development and validation. Details of the tests are summarized in the Supplementary Sect. 4.

**Table 2:** Comparison of conventional and automated methods of [^18^F]FBB quality control testing.

QC test	Conventional method	Tracer-QC method
Color	Visual assessment	Absorbance measurement (with disposable indicators)
Clarity	Visual assessment	
pH	Indicator + visual assessment	
Residual Kryptofix	Spot test + visual assessment	
Endotoxin concentration	Portable test system (PTS) reader	
Residual solvents	Gas chromatograph	
Radionuclidic identity (half-life)	Dose calibrator + clock	Emission measurement (with disposable scintillators)
Radioactivtiy concentration	Dose calibrator + syringe	
Radiochemical identity/purity	Stand-alone radio-HPLC	Radio-HPLC integrated in Tracer-QC supported by a disposable kit
Chemical identity/purity		
Molar activity		

### Ethics approval

This article does not contain any studies with human participants or animals performed by any of the authors.

## Results

### [^18^F]FET production and testing

In initial synthesis runs with < 20 MBq starting activity, the automated droplet synthesis exhibited very good 70 ± 9% (n = 9) crude RCY. Notably, this was higher than the previously reported manual droplet-based synthesis (59 ± 7%, n = 4)^43^ or automated results using the passive-transport droplet-based synthesizer (54 ± 6%, n = 5)^43^. Additionally, the system had an improved synthesis time of 18 min compared to 24 min or 19 min for manual or passive transport automated system, respectively. Detailed comparison of various parameters is shown in Supplementary Table S2. Previous work with [^18^F]fallypride showed similar improvements when transitioning from the passive-transport (PT) chip to the STT chip^42^.

**Figure 5:**
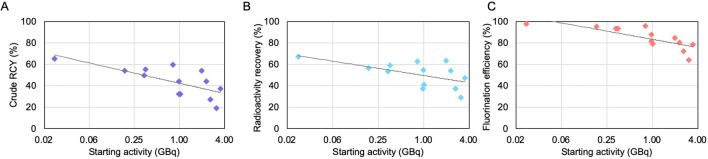
Performance of crude [^18^F]FET droplet-based radiosynthesis as a function of starting activity. (**A**) Crude RCY. (**B**) Radioactivity recovery. (**C**) Fluorination efficiency. Note that the x-axis is plotted on a logarithmic scale, and a logarithmic trendline is generated for all graphs.

The impact of increased starting activity on the performance of the crude synthesis was also explored (Fig. 5). Crude RCY decreased from ~ 70% to ~ 40% as activity was increased in the range 0.2–4 GBq. The crude RCY is a product of radioactivity recovery and fluorination efficiency and both these parameters show a slight decrease with increased starting activity. A similar result was previously observed with [^18^F]fallypride synthesis^33^.

**Figure 6:**
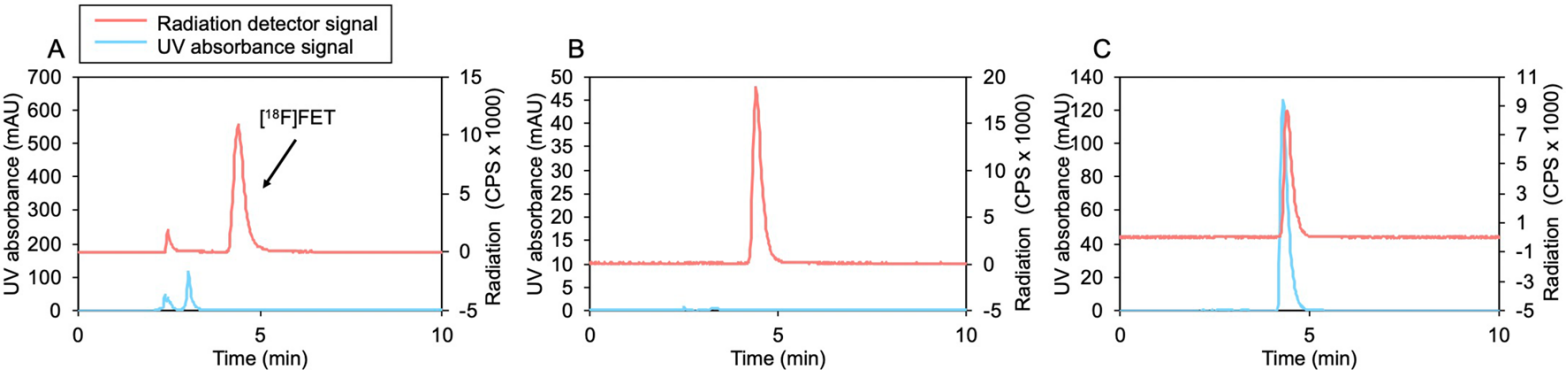
Example HPLC chromatograms for [^18^F]FET. (**A**) Crude product. (**B**) Formulated product. (**C**) Formulated product co-injected with reference standard.

The overall synthesis time, including purification and formulation, was 60 min. For clinical-scale batches, the synthesis exhibited 28 ± 14% (n = 3) overall activity yield, > 99% radiochemical purity, and high molar activity (418 ± 52 GBq/µmol, n = 3; EOS). Three consecutive batches of formulated [^18^F]FET passed QC tests (Supplementary Table S3), with most impurities being below detectable limits or extremely low. Example chromatograms during [^18^F]FET purification and assessment of radiochemical purity and identity are shown in Fig. 6.

### [^18^F]FBB production and testing

The initial runs using low (< 20 MBq) starting activities were performed for syntheses with 2 different precursor volumes (10 µL and 15 µL). The crude RCY was similar in both cases (54 ± 9%, n = 5 for 15 µL and 58 ± 7%, n = 6 for 10 µL) as were other parameters (Supplementary Table S4). Using a larger precursor volume helped to increase tolerance to any dispensing errors that may occur due to the high viscosity of the precursor solution.

**Figure 7:**
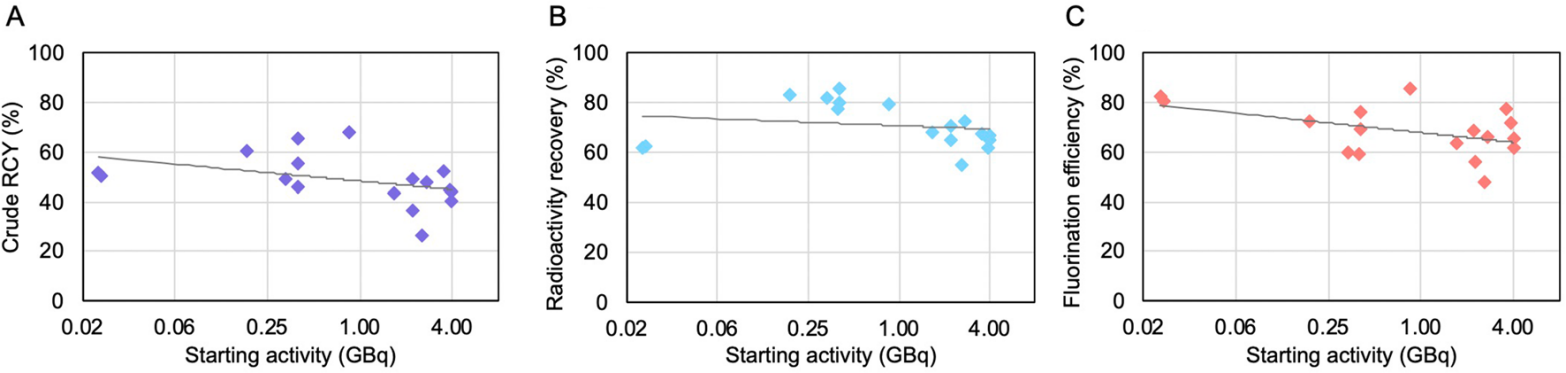
Performance of crude [^18^F]FBB droplet-based radiosynthesis as a function of starting activity. (**A**) Crude RCY. (**B**) Radioactivity recovery. (**C**) Fluorination efficiency. Note that the x-axis is plotted on a logarithmic scale, and a logarithmic trendline is generated for all graphs.

The impact of starting activity on the synthesis performance was also investigated (Fig. 7). Across the range of 0.02–4.0 GBq, the crude RCY exhibited a slight decrease, though the impact was negligible up to ~ 1 GBq of starting activity. Both the component measurements radioactivity recovery and fluorination efficiency exhibited a similar trend.

**Figure 8:**
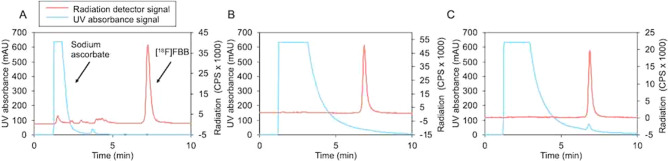
Example HPLC chromatograms for [^18^F]FBB. (**A**) Crude product. (**B**) Formulated product. (**C**) Formulated product co-injected with reference standard.

Complete tracer production—microdroplet synthesis followed by analytical HPLC purification and automated SPE formulation—took ~ 60 min and resulted in a radiochemically pure (> 95%) product. Three consecutive batches exhibited 15 ± 4% (n = 3) overall activity yield, and high molar activity 480 ± 190 GBq/µmol (n = 3; EOS). All batches passed necessary QC tests (Supplementary Table S5). Example chromatograms during [^18^F]FBB purification and assessment of radiochemical purity and identity are shown in Fig. 8.

Another three consecutive batches were prepared for QC analysis with the Tracer-QC system. This set of runs exhibited overall activity yield of 16 ± 4% (n = 3) and molar activities of 490 ± 130 GBq/µmol (n = 2; EOS). Automated testing of each batch was followed by automated analysis producing a summary page along with a detailed 26-page report. All samples passed all acceptance criteria for release of the doses, with many impurities below detection limits. The acceptance criteria for [^18^F]FBB QC, along with measured results for each batch are summarized in Supplementary Table S6. The demonstration with 3 consecutive samples confirms consistency of both the synthesis and QC testing.

## Discussion

### Comparison to conventional synthesis

We previously showed, for the syntheses of [^18^F]FET^43^ and [^18^F]FBB^45^, that miniaturization of radiopharmaceutical production leads to many benefits compared to conventional synthesis, including reduced reagent consumption, shorter reaction time, high molar activity, and high reaction yields, on top of the very small physical footprint of the microfluidic system. In this work the synthesis activity scale is increased up to 4 GBq with minimal modifications to the synthesis parameters. The precursor consumption remained low, consuming 100–150 × less than macroscale methods. With higher starting activities the synthesis time is slightly longer, due to the need to dry a larger volume of the radioisotope solution, but still remains < 60 min (conventional reported synthesis times vary between 45 and 90 min). The yields are comparable to the range reported for conventional methods. Here, [^18^F]FET was produced with 36 ± 7% (n = 3) overall yield and generally, for conventional syntheses the reported yields vary between 20 and 40%^46,47,50^. Microdroplet [^18^F]FBB synthesis resulted in 23 ± 3% (n = 6) overall yield comparable to 10–30% yield range of most reported methods^48,51–54^. As expected, the molar activity of the microscale synthesis remained high (> 400 GBq/µmol) at the increased activity scale.

### Activity scaling in droplet micro-radiosynthesizer

In previous work by our group, droplet-based synthesis of [^18^F]fallypride was demonstrated with starting activities ranging up to 41 GBq^33^, highlighting the scalability of the droplet radiosynthesis techniques. Up to 7.2 GBq of injectable [^18^F]fallypride was produced, which would be sufficient for multiple clinical doses. This work further demonstrates that product amounts of additional clinically-relevant radiotracers ([^18^F]FET and [^18^F]FBB) can be scaled up to amounts sufficient for clinical PET scans.

For [^18^F]fallypride, the concentration of aqueous fluoride-18 was performed using a custom micro-cartridge-based radioisotope concentrator that could reduce the volume from several mL to less than 30 µL in under 8 min^39^. However, this and other cartridge-based concentration approaches add complexity to the overall synthesizer setup. In this work, the starting [^18^F]fluoride activity was scaled (up to 4 GBq) by directly loading and drying multiple 30 µL droplets of the [^18^F]fluoride solution (without using a cartridge or additional valves). Another significant advantage of this concentration method is that it can be used with any amount of base (in contrast to cartridge concentration methods, in which the type and amount of base is linked to the elution efficiency). The independence of the approach here is that one can ensure that the total amount of base added with the [^18^F]fluoride matches the optimal amount of base in the reaction as determined from low-activity optimization studies. While it is possible to load even higher activities than reported here (i.e., > 4 GBq) with this method, drying a large volume (e.g. 1 mL) would require many (33) droplets to be sequentially loaded and dried. With each evaporation cycle run for 1.5 min, drying of 1 mL would take approximately 50 min. We expect ~ 300–600 µL to be an upper practical limit, which could be concentrated in 15–30 min, though for many applications, smaller volumes and activity levels would be sufficient. For example, the concentration of 100 µL could be completed in < 6 min, which can contain 4 GBq or more of activity, depending on target volume and bombardment parameters. In this work ~ 100 µL of [^18^F]fluoride (~ 2–4 GBq) afforded 0.4–0.7 GBq of injectable tracer, which is sufficient for a typical clinical PET scan (~ 0.37 GBq per injection). Overall, sequential drying results in a significantly simpler procedure and more compact synthesis system compared to cartridge-based methods^33^.

At the same time as we are attempting to increase the activity scale of the synthesis, improvements in scanner technology are requiring less activity for clinical PET scans. In particular, recent developments with total-body PET allowed good human [^18^F]FDG scans to be obtained with only 25 MBq of administered activity^55^, about ~ 10 × lower than what is typically injected. Such advancements mean that in the future the modest sized batches produced here may each be suitable for many patients, or batches for one or a few patients could be produced with lower starting activity levels.

### Impact of starting activity on synthesis performance

Increasing starting radioactivity in radiopharmaceutical syntheses can directly affect the stoichiometry of a reaction and amplify radiolysis effects. We observed that the reaction performance was relatively unaffected up to ~ 1 GBq starting activity, and then started to show some reduction for both [^18^F]FET and [^18^F]FBB. Both the fluorination efficiency and radioactivity recovery exhibited some decline, suggesting reduced fluoride-18 incorporation and resulting in moderately lower crude RCY. In previous work with [^18^F]fallypride, the decrease in crude RCY only became significant around 20 GBq^33^, indicating that this effect may vary between different syntheses. Interestingly, for a microvial-based synthesis of [^18^F]FET in 10 µL volume by Iwata et al*.*, the reaction yield was reported constant when starting activity was varied between 0.1 and 6 GBq^31^. However, upon addition of fluoride-19 carrier (simulating a further increase in activity), the RCY was reduced significantly^31^. Looking at the work by Iwata et al*.* and the current reported results of [^18^F]FET syntheses, a higher activity may have been better tolerated in the first case due to the higher amount of precursor used (180 nmol, compared to 60 nmol in our work), or differences in purity of the [^18^F]fluoride source (i.e. the [^18^F]fluoride undergoes cartridge trap and release process while in our case it is used directly from a cyclotron). Overall, impurities in the fluoride-18 solution, reduced excess of precursor, and radiolysis are all potential culprits for the observed reduction in the reaction yields with higher starting activities. Further studies are needed to fully understand these effects and improve reaction scalability in the future.

### Quality control testing

After synthesis, purification, and formulation, quality control (QC) testing of the radiopharmaceuticals is a crucial step necessary to ensure safety prior to use in patients^56–61^. In this work we performed QC testing both using conventional procedures as well as a new automated QC testing platform (Tracer-QC). In general, likely due to the small total amounts of solvents and reagents, the amounts of impurities were extremely low, suggesting that microvolume methods may offer some inherent safety advantages for radiopharmaceutical production. Conventional QC tests require an array of expensive analytical instrumentation, all of which requires space, maintenance, training, calibration, and documentation, making such testing a time-consuming, expensive procedure^49,62^. Furthermore, some of the tests require manual handling of the radioactive batches resulting in high radiation exposure to the operator^63^ and higher margin for human error or subjective interpretation. Moreover, pairing a compact microfluidic reactor system with a large analytical laboratory facility undermines the economic and practical advantages offered by microfluidic technology. In contrast, the compact and automated Tracer-QC system with integrated HPLC^49^ offers key advantages which allow to overcome these challenges. (1) Ease and safety of use. Unlike conventional test methods that require expertise in operation and maintenance of many different analytical instruments, the integrated platform requires only a simple setup and operating procedure with minimal need for training. It also avoids the need for subjective assessments of test results, reducing variability and preventing human error. Safety is significantly improved because there is never a direct line of sight between the user and unshielded sample, and minimal user interaction with the system is needed. The instrument is also very easy to maintain due of its simplicity, absence of cleaning and the large number of automated internal diagnostics. (2) Efficiency. Because Tracer-QC runs completely unattended, personnel are freed up to perform other tasks after setup and initiation of tests. All necessary QC tests for [^18^F]FBB batches were carried out completely unattended, and the software automatically generated a detailed report with “pass/fail” results for all QC tests. The automated suitability checks and calibrations further reduce the operator effort. Additional efficiencies arise due to the compact size of the system, minimizing the laboratory space dedicated to QC testing. The kit-based design minimizes effort to maintain the consumables inventory and supports the production of multiple different radiopharmaceuticals daily by a single Tracer-QC system.

## Conclusion

In this work we demonstrate the use of a compact automated microdroplet synthesizer to rapidly produce batches of formulated [^18^F]FET and [^18^F]FBB with high yield and high molar activity. In contrast to previously reported production of [^18^F]fallypride on a microdroplet chip which was coupled to a separated radionuclide concentrator to increase the synthesis scale^33^, the radioisotope was concentrated in this work using a simpler and faster approach still capable of clinically-relevant synthesis scale. Though a modest reduction in RCY was observed when scaling up, it is nonetheless clear that droplet-based radiochemistry systems have sufficient scaling capacity to produce batches for one or multiple clinical doses (that pass clinical quality control tests), while offering advantages such as compact size, reduced reagent usage, high molar activity and fast synthesis time^19^. Because employing conventional approaches to perform QC testing seriously undermines the potential of miniaturized synthesizers, in this work we demonstrate an alternative approach. Pairing of the droplet synthesizer with an automated benchtop QC testing system (Tracer-QC) has the potential to establish a robust, rapid, compact and economical method for batch-on-demand production of PET radiopharmaceuticals, without requiring large radiochemistry and analytical chemistry facilities.

## Supplementary Information

Supplementary Information.

## Data Availability

Most of the experimental data is reported in the manuscript and in the supplemental information. The additional datasets for each individual experiment are available from the corresponding author on reasonable request.
