# Supplementary material for: Economical droplet-based microfluidic production of [18F]FET and [18F]Florbetaben suitable for human use
Source: Sci Rep. 2021 Oct 19;11:20636. doi: 10.1038/s41598-021-99111-4 (PMC8526601; doi:10.1038/s41598-021-99111-4)
Supplement: Supplementary file 1 — Supplementary Information. [file 41598_2021_99111_MOESM1_ESM.pdf]

Supplemental information

Economical droplet-based microfluidic production of [^{18}F]FET and [^{18}F]Florbetaben suitable for human use

Ksenia Lisova* [1,2,3], Jia Wang* [1,2,4], Tibor Jacob Hajagos[4], Yingqing Lu[1,2,3], Alexander Hsiao[6], Arkadij Elizarov[5], R. Michael van Dam[1,2,3,4]
*Equal contribution
[1]Crump Institute for Molecular Imaging, University of California Los Angeles, Los Angeles, CA, USA
[2]Department of Molecular & Medical Pharmacology, University of California Los Angeles, Los Angeles, CA, USA
[3]Physics in Biology and Medicine Interdepartmental Graduate Program, University of California Los Angeles, Los Angeles, CA, USA
[4]Bioengineering Department, University of California Los Angeles, Los Angeles, CA, USA
[5]Trace-Ability, Inc., Van Nuys, CA, USA
[6]SOFIE, Inc., Culver City, CA, USA

Contents

1 Droplet synthesizer setup 2
2 Analytical methods (radio-TLC, radio-HPLC) 2
3 Quality control testing methods (conventional instruments) 3
4 QC testing with Tracer-QC 4
5 [^{18}F]FET synthesis (low activity) 6
6 [^{18}F]FET quality control results 8
7 [^{18}F]FBB synthesis (low activity) 9
8 [^{18}F]FBB quality control results (conventional) 10
9 [^{18}F]FBB quality control results (Tracer-QC) 11

1 Droplet synthesizer setup

During setup of the automated microvolume synthesis system, reagents were loaded into the dispensers as shown in **Table S1**. Dispensers were primed before use.

	**[^{18}F]FET**	**[^{18}F]FBB**
**Dispenser 1**	[^{18}F]fluoride / $TBAHCO_3$	[^{18}F]fluoride / K_{222} / K_2CO_3
**Dispenser 2**	FET precursor solution	FBB precursor solution
**Dispenser 3**	FET deprotection solution	FBB deprotection solution
**Dispenser 4**	FET collection solution	FBB collection solution

Table S1: Reagent setup in automated droplet synthesizer for syntheses of [^{18}F]FET and [^{18}F]FBB.

2 Analytical methods (radio-TLC, radio-HPLC)

Fluorination efficiency was determined via radio-thin-layer chromatography (radio-TLC). For [^{18}F]FET, silica gel 60 F254 plates (Merck KGaA, Darmstadt, Germany) were cut into 15 x 60 mm pieces (with 40 mm developing distance), spotted with 0.5 µL of the sample and developed in 80% (v/v) MeCN in H_2O. TLC plates were analyzed with a Cerenkov luminescence imaging system as previously described [49]. Retention factors of the observed radioactive species were: 0 ([^{18}F]fluoride), 0.3 ([^{18}F]FET), and 0.8 (fluorinated intermediate). For [^{18}F]FBB, reverse phase TLC plates (RP-18 silica gel 60 F254 sheets; aluminum backing; Millipore Sigma, St. Louis, MO, USA) were prepared and used in a similar fashion, but developed in 90% (v/v) MeCN in H_2O. Retention factors of the observed radioactive species were: 0.0 ([^{18}F]fluoride), 0.4 ([^{18}F]FBB), and 0.8 (fluorinated intermediate).

Radio-HPLC analysis and purification were performed on an analytical-scale Smartline HPLC system (Knauer, Berlin, Germany) with 200 µL injection loop, a pump (Model 1000), degasser (Model 5050), UV detector (Model 2500) and a radiometric detector (Bioscan B-FC-4000, Bioscan Inc., Washington DC, USA). Samples were separated using a C18 column (Luna, 5 µm particles, 100Å pores, 250 x 4.6 mm, Phenomenex, Torrance, CA, USA) with guard column (SecurityGuard C18, Phenomenex). For [^{18}F]FET, separation was performed isocratically using 10% (v/v) EtOH in H_2O at a flow rate of 1 mL/min, and UV absorbance was measured at 269 nm. The retention time of [^{18}F]fluoride was ~2-3 min, and ~5 min for [^{18}F]FET. The fluorinated intermediate and other impurities were eluted off the column by switching the mobile phase to 95:5 (v/v) MeCN:H_2O. For [^{18}F]FBB, the mobile phase was 60:40 (v/v) MeCN : 25 mM phosphate buffer at a flow rate of 1.5 mL/min, and UV absorbance was measured at 254 nm. The observed retention times were ~2-3 min for [^{18}F]fluoride, 6 min for [^{18}F]FBB, and 14 min for the fluorinated intermediate.

3 Quality control testing methods (conventional instruments)

Quality control tests for appearance, pH, radionuclide purity and identity, bacterial endotoxins, sterility, radiochemical and chemical purity were determined as previously described [1].

Molar activity

Molar activity was estimated by quantifying amount of the tracer in purification chromatogram using ultraviolet (UV) peak and cold standard calibration curve, then dividing by radioactivity of the isolated product after purification.

Residual content of $TBAHCO_3$

Residual TBAHCO3, which has acceptable limit of 2.6 mg/V, in the purified sample was determined using a thin-layer chromatography (TLC) spot test method reported by Kuntzsch et al [2]. For 5 mL formulation volume the calculated limit would be 520 mg/L, however the expected quantity of $TBAHCO_3$ would be much less. Thus a low concentration standard solution of $TBAHCO_3$ (45 mg/L) was created and spotted alongside the formulated [^{18}F]FET (2 µL) onto a silica TLC plate (JT4449-2, J.T. Baker, Center Valley, PA, USA), and air dried. 10 µL of a developing solution (0.72M NH4OH in 90% MeOH) was added on top of each spot, dried, and then the TLC strip was developed in a chamber containing iodine crystals for 1 min. The color intensity of the spot of the purified sample was compared to that of the standard solution to confirm the residual amount was below the injectable limit.

Residual content of K_{222}

Residual kryptofix content was determined using a TLC spot test as reported by Halvorsen et al [3]. Iodoplatinated TLC strips were prepared according to the reported procedure. The standard solutions containing 50 µg/mL (injectable limit) and 12.5 µg/mL of kryptofix in a formulation matrix identical to [^{18}F]FBB formulation matrix were prepared. 2 µL of [^{18}F]FBB sample was spotted alongside the standards onto a iodoplatinated TLC strip, the spots were air dried followed by addition of 1% H_2O_2 (2.5 µL). After 1 min of drying the sample spots were analyzed for K_{222} content.

Residual solvent analysis

The concentration of residual solvents (i.e. methanol, acetonitrile, thexyl alcohol, ethanol and DMSO) was determined using gas chromatography mass spectrometry (GCMS).

Residual solvent analysis of [^{18}F]FET samples

Gas chromatography mass spectrometry (GC-MS) measurements were carried out on a GC system (6890N, Agilent) equipped with mass spectrometry detector (5975 MSD) and autosampler (7683B). The instrument was controlled by Enhanced Chemstation software version E.01. The inlet was operated in split mode at 250 °C. Ultra-high purity He (Airgas West, Culver City, CA) was used as the carrier gas with the flowrate set to 1.2 mL / min. Separation was carried out on a 30 m x 250 µm x 0.25 µm DB-Wax column (Agilent J&W). The GC oven was initially held at 70°C, heated to 140 °C at 10 °C/min, and then heated to 260 °C at 30 °C/min. The MSD was operated in the scan mode and used EI ionization.

Instrument response for known concentrations of pure analytes in butanol was measured to determine the analyte concentrations in the samples. More specifically, a 4-point calibration curve was generated for all solvents (MeOH, MeCN, TA, EtOH). The concentration of the residual analytes was then interpolated from this calibration curve.

Residual solvent analysis of [^{18}F]FBB samples

The concentrations of residual solvents (i.e. acetonitrile, DMSO, ethanol) were determined using headspace gas chromatography mass spectrometry (GCMS). To 100 µL of each sample, 1 µL of $^{2}H_6$-DMSO was added as an internal standard. For acetonitrile and DMSO, an aliquot (10 µL) of each sample was transferred to 10 mL glass headspace vials fitted with magnetic caps. For measurement of ethanol concentrations, the samples were diluted 1 to 100 with water prior to the transfer. Samples were incubated for 20 min at 200 °C with gentle agitation every 10 seconds. After incubation, 1 mL of headspace vapor was withdrawn with a heated (110 °C) syringe and injected onto a GC inlet (1/10 split, 250 °C). Ultra-high purity He (Airgas West, Culver City, CA) was used as the carrier gas at constant flow (1 mL/min). Separations were carried out on a bonded-phase non-polar fused silica capillary column (60 m x 250 µm x 0.25 µm Zebron ZB-5plus column, Phenomenex). The GC oven was initially held at 50°C for 2 min, then was heated to 250 °C at 10 °C/min. The end of the column (GC/EI-MS transfer line at 250°C) was inserted into the EI source (200°C, 70 eV) of a high resolution Orbitrap mass spectrometer (Thermo Scientific Q Exactive GCMS, calibrated with perfluorotributylamine immediately prior to the analysis of each batch of samples), scanning from m/z 30-500 at a resolution (FWHM) of 60,000. Data were collected with instrument manufacturer-supplied software (Thermo Xcalibur v4.1). Instrument response from known concentrations of pure analytes in PEG400/water mixtures containing the same amount of internal standard was measured to determine the analyte concentrations in the samples. More specifically, a five-point calibration curve was generated for all three solvents at the following concentration levels: 0, 25.625, 51.25, 102.5, and 205 PPM for acetonitrile; 0, 312.5, 325, 1250, 2500 PPM for DMSO; and 0, 3.75, 7.5, 15, and 30% (w/v) for ethanol. Calibration curves for acetonitrile and ethanol were constructed by directly comparing absolute peak area (ordinate) and solvent concentration (abscissa). On the other hand, for the DMSO calibration curve, ratios of DMSO/$^{2}H_6$-DMSO peak areas were used as the ordinate to account for potential sulfoxide oxidation.

4 QC testing with Tracer-QC

The Tracer-QC system uses optical measurements for all non-chromatographic QC tests. For color and clarity, the signal is the spectrophotometric measurement of absorbance of light passed through the sample in the plate reader. For pH, kryptofix, endotoxin and acetonitrile, the sample's interaction with an indicator (contained in the disposable kit) designated for each of the tests (and mixed with sample by the liquid handler) leads to unique changes in the absorbance spectrum. For radionuclidic identity and radioactivity concentration, the signal is a luminescence measurement detecting the emission of light from scintillating materials that interact with the radioactive sample in the kit. For the HPLC group of tests the signals are the UV and radio-chromatograms generated by traditional HPLC detectors. The Tracer-QC software

processes the obtained signals in the context pf pre-set parameters and measurements from reference standards (contained in the disposable kit) to determine the values of all QC parameters. Each test has automated suitability checks which confirm whether the produced measurement is valid. After values have been calculated and verified for all QC tests, the software produces a comprehensive report with these values along with acceptance criteria and pass/fail determination. These principles allow the entire QC process to be automated and objective while supporting completely traceable and tamper-free data flow from raw measurements to the report.

Color
A spectrophotometric measurement of the sample is performed together with a positive control solution containing one or more color standards with known absorbance.

Clarity
A turbidimetric analysis of the sample along with positive and negative control solutions is conducted through spectrophotometric measurements.

pH
The solution to be analyzed is mixed with an indictor solution, which produces a pH-dependent change in the indicator's absorbance spectrum within the sample and indicator mixture.

Bacterial endotoxin
Enzymatic activation of serine proteases from horseshoe crab amebocyte lysate by interaction with bacterial endotoxin is used to produce a chromogenic signal that can be analyzed spectrophotometrically.

Radioactivity concentration
The radioactivity of an aliquot of sample solution is determined from the intensity of its radioluminescent emission.

Radionuclidic identity (half-life)
The time-dependent radioactivity of an aliquot of sample solution is determined from the intensity of its radioluminescent emission.

Chemical identity, chemical purity, and/or chemical content via HPLC
While chemical identity, chemical purity, and chemical content are all separate properties that each have distinct meaning and corresponding product specification, in common practice they can be derived from the same experiment simultaneously in cases where a product specification calls for the determination of more than one. In addition, these tests can be carried out concomitantly with determination of radiochemical identity, radiochemical impurity, and/or specific activity. For the Tracer-QC platform, all liquid handling required for sample preparation and injection is handled by the pipetting robot, mated to a conventional HPLC system utilized to set flow rates and/or gradients and detect elution of compounds.
Radiochemical identity, radiochemical purity, and/or molar activity via Radio-HPLC

The radiochemical identity and radiochemical purity, and molar activity tests can be carried out concomitantly with determination of chemical identity, and/or chemical purity. Molar activity is then derived from a combination of the chemical content and radiochemical purity measurements (via HPLC) and radioactivity concentration.
The Tracer-QC HPLC method (**Figure S1**) is an adaptation of the validated HPLC method for analysis of Neuroceq formulation. For the blank injection, Neuroceq formulation matrix is injected directly with no dilution steps. For quantification of analyte peaks, a reference standard solution of [^{19}F]FBB (1.5 µg/mL), Stb-OMs (1.25 µg/mL), and Boc-Stb-TEG (2.5 µg/mL) in acetonitrile is also injected directly with no dilution steps. In order to minimize the volume of sample dose required for the TA-FBB-HPLC test, the sample dose is diluted in a 1:4 ratio with water before injection.

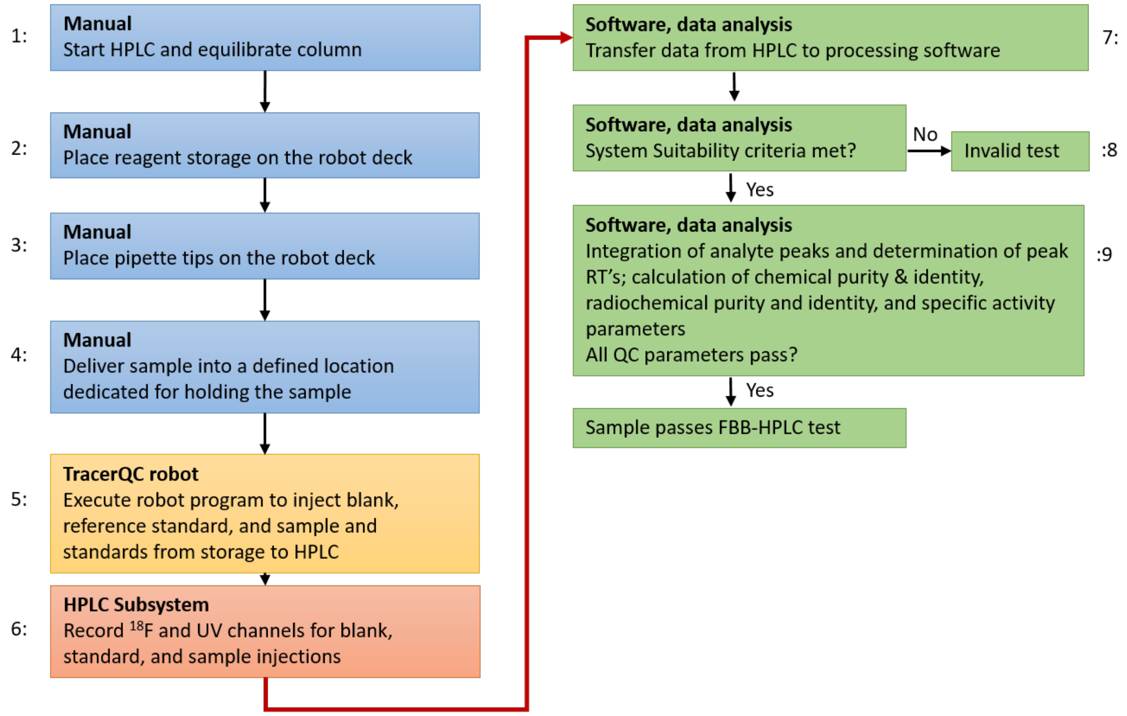

Figure S1: Tracer-QC HPLC measurement protocol

Once the blank, standard, and sample dose injections have been completed, integration of the analyte peaks is performed, and data processing software calculates the values of all parameters required for release testing.

5 [^{18}F]FET synthesis (low activity)

Table S2 summarizes [^{18}F]FET synthesis performance at low starting activity (<20 MBq) and compares the results to previous work using manual operation of a simplified droplet reaction chip [4] and automated operation of a passive-transport reaction chip [4]. For manual operation, this chip was mounted on a similar heater, but reagents were delivered with a micropipette and product was collected with a micropipette.

	Surface-tension trap chip (this work)	Surface-tension trap chip	Passive-transport chip
**Synthesis operation**	Automated	Manual	Automated
**Number of replicates (n)**	9	4	5
**Radioactivity recovery (%)**	80 ± 6	64 ± 5	59 ± 10
**[^{18}F]FET conversion (%)**	88 ± 7	92 ± 4	93 ± 6
**Synthesis time (min)**	18	24	19
**Crude RCY (%)**	70 ± 9	59 ± 7	54 ± 6
**Residual activity on chip (%)**	0.7 ± 0.6	1.3 ± 0.5	3.2 ± 1.5

Table S2: Performance of droplet-based [^{18}F]FET synthesis on several platforms.

6 [^{18}F]FET quality control results

Test	Testing Criteria	Batch 1	Batch 2	Batch 3
Appearance	Clear, colorless, particle free	Pass	Pass	Pass
Radioactivity concentration	7.4-74 MBq/mL [0.2-2 mCi/mL]	47 MBq/mL [1.3 mCi/mL]	56 MBq/mL [1.5 mCi/mL]	46 MBq/mL [1.3 mCi/mL]
Molar Activity	> 37 GBq/µmol [1 Ci/ µmol]	420 GBq/µmol [11.4 Ci/µmol]	697 GBq/µmol [18.8 Ci/µmol]	595 GBq/µmol [16.1 Ci/µmol]
Radiochemical identity	Retention time ratio of radio peak vs cold standard (0.9-1.1)	1.0	1.0	1.0
$TBAHCO_3$	<520 mg/L*	< 45 mg/L	< 45 mg/L	< 45 mg/L
Residual solvents	MeCN < 410 PPM<br>MeOH < 3000 PPM<br>TA < 5000 PPM<br>EtOH < 10%	N.D.<br>N.D.<br>N.D.<br>N.D.	N.D.<br>N.D.<br>N.D.<br>N.D.	N.D.<br>N.D.<br>N.D.<br>N.D.
Radiochemical purity	> 95%	> 99%	> 99%	> 99%
Radionuclide identity	104-115 min	109	108	110
pH	4.0-7.0	5.0	5.5	5.5
Filter integrity	> 50 PSI	> 50 PSI	> 50 PSI	> 50 PSI
Shelf life	Pass appearance, pH and radiochemical purity after 240 min	Pass	Pass	Pass
Gamma ray emission energy	496-526 keV photons	Pass	Pass	Pass
Radionuclide purity	No less than 99.5%	Pass	Pass	Pass
Bacterial endotoxin	< 175 EU/total batch	Pass	Pass	Pass
Sterility	No colony growth observed for 14 days	Pass	Pass	Pass

Table S3: Conventional (manual) quality control testing results for 3 consecutive batches of [^{18}F]FET. *Acceptable limit is calculated based on < 2.6 mg/V regulation where V is a total maximum injection volume, in this case we compute for 5 mL as total formulation volume. N.D. = not detected. Limits of detection for residual solvents are: 40 ppm for MeCN , 30 ppm for MeOH, 40 ppm for TA, 50 ppm for EtOH.

7 [^{18}F]FBB synthesis (low activity)

Table S4 summarizes [^{18}F]FBB synthesis performance at low starting activity (<20 MBq) on the surface tension trap chip, and compares the effect of using 10 or 15 µL of precursor stock solution.

	**Automated**	**Automated**	**Manual**
Precursor volume (µL)	15	10	10
Number of replicates (n)	5	6	4
Radioactivity recovery (%)	63 ± 6	69 ± 9	66 ± 6
[^{18}F]FBB conversion (%)	86 ± 9	86 ± 9	96 ± 1
Synthesis time (min)	18	18	23
Crude RCY (%)	54 ± 9	58 ± 7	63 ± 6
Residual activity on chip (%)	7 ± 6	8 ± 4	1 ± 1

Table S4: A comparison of droplet-based [^{18}F]FBB synthesis performance when performed manually versus automated, and at 2 different precursor solution volumes.

8 [^{18}F]FBB quality control results (conventional)

Test	Testing Criteria	Batch 1	Batch 2	Batch 3
Appearance	Clear, colorless, particle free	Pass	Pass	Pass
Radioactivity concentration (MBq/mL)	45-5000	83 MBq/mL [2.2 mCi/mL]	97 MBq/mL [2.6 mCi/mL]	151 MBq/mL [4.1 mCi/mL]
Molar Activity (GBq/μmol)	> 37	593 GBq/μmol [16.0 Ci/μmol]	262 GBq/μmol [7.1 Ci/μmol]	583 GBq/μmol [15.7 Ci/μmol]
Radiochemical identity	Retention time ratio of radio peak vs cold standard (0.9-1.1)	1.0	1.0	1.0
K_{222}	< 50 mg/L	< 13 mg/L	< 13 mg/L	< 13 mg/L
Residual solvents	MeCN < 410 PPM<br>DMSO < 5000 PPM<br>Ethanol < 15%	N.D.*<br>529 PPM<br>8%	N.D.<br>218 PPM<br>7%	N.D.<br>229 PPM<br>7%
Radiochemical purity	> 95%	97%	98%	98%
Radionuclide identity	105-115 min	113	112	113
pH	4.0 - 8.0	5.5	5.5	5.5
Filter integrity	> 50 PSI	> 50 PSI	> 50 PSI	> 50 PSI
Shelf life	Pass appearance, pH and radiochemical purity after 240 min	Pass	Pass	Pass
Gamma ray emission energy	496-526 keV photons	Pass	Pass	Pass
Radionuclide purity	No less than 99.5%	Pass	Pass	Pass
Bacterial endotoxin	< 175 EU/total batch	Pass	Pass	Pass
Sterility	No colony growth observed for 14 days	Pass	Pass	Pass

Table S5: Conventional (manual) quality control testing results for 3 consecutive batches of [^{18}F]FBB. N.D. = not detected. Limit of detection for MeCN is 20 ppm.

9 [^{18}F]FBB quality control results (Tracer-QC)

The report of the QC testing performed on additional 3 consecutive batches of [^{18}F]FBB with an automated Tracer-QC unit are shown in **Table S6.** Note that the indicated values for concentration and molar activity are slightly lower than would be expected in practice due to the decay that occurred during transport of samples from UCLA to Trace-Ability (~30 min) prior to starting the QC tests.

Test Parameter	Specification	Batch #1	Batch #2	Batch #3
Color (mAU)	< 500	98.2	43.7	91.8
Clarity (NTU)	< 10	8.0	7.3	9.9
pH	4.5 – 7.5	5.2	5.3	5.3
Endotoxin (EU/mL)	< 7.5	< 1	< 1	< 1
Acetonitrile (µg/mL)	< 410	< 100	< 100	< 100
Kryptofix (µg/mL)	< 50	< 50	< 50	< 50
Concentration (GBq/mL)	45 – 5000	285	244	400
Half-Life (min)	105 – 115	110.7	113.2	114.6
Chemical Identity (%RRT)	90 – 110	100.0	100.0	100.0
trans-FBB Content (µg/mL)	≤ 3.0	0.53	0.62	0.78
Stilbene-OMs Content (µg/mL)	≤ 3.0	0.00	0.00	0.08
BOC-Stilbene-TEG Content (µg/mL)	≤ 1.5	0.06	0.11	0.00
Unspecified Impurity Content (µg/mL)	≤ 5.0	0.00	0.00	0.00
trans-FBB Radiochemical Identity (%RRT)	90 – 110	100.0	100.0	100.0
cis-FBB Radiochemical Identity (RRT)	1.12 – 1.16	Not Detected	Not Detected	Not Detected
cis/trans-FBB Radiochemical Purity (%)	≥ 93	96.6	95.4	96.4
cis-FBB Radiochemical Content (%)	≤ 6%	0.0	0.0	0.0
Unspecified Radiochemical Impurity (%)	≤ 7%	3.4	4.6	3.6
Molar activity (GBq/µmol)	≥ 3	196.0	142.9	185.3

Table S6: Tracer-QC (automated) quality control testing results for 3 consecutive batches of [^{18}F]FBB.
